# Gene networks and metabolomic screening analysis revealed specific pathways of amino acid and acylcarnitine profile alterations in blood plasma of patients with Parkinson’s disease and vascular parkinsonism

**DOI:** 10.18699/vjgb-24-100

**Published:** 2024-12

**Authors:** A.A. Makarova, P.M. Melnikova, A.D. Rogachev, P.S. Demenkov, T.V. Ivanisenko, E.V. Predtechenskaya, S.Y. Karmanov, V.V. Koval, A.G. Pokrovsky, I.N. Lavrik, N.A. Kolchanov, V.A. Ivanisenko

**Affiliations:** Institute of Cytology and Genetics of the Siberian Branch of the Russian Academy of Sciences, Novosibirsk, Russia Novosibirsk State University, Novosibirsk, Russia; Novosibirsk State University, Novosibirsk, Russia; Novosibirsk State University, Novosibirsk, Russia N.N. Vorozhtsov Novosibirsk Institute of Organic Chemistry of the Siberian Branch of the Russian Academy of Sciences, Novosibirsk, Russia; Institute of Cytology and Genetics of the Siberian Branch of the Russian Academy of Sciences, Novosibirsk, Russia Novosibirsk State University, Novosibirsk, Russia Kurchatov Genomic Center of ICG SB RAS, Novosibirsk, Russia; Institute of Cytology and Genetics of the Siberian Branch of the Russian Academy of Sciences, Novosibirsk, Russia Novosibirsk State University, Novosibirsk, Russia Kurchatov Genomic Center of ICG SB RAS, Novosibirsk, Russia; Novosibirsk State University, Novosibirsk, Russia; Institute of Cytology and Genetics of the Siberian Branch of the Russian Academy of Sciences, Novosibirsk, Russia Novosibirsk State University, Novosibirsk, Russia; Institute of Chemical Biology and Fundamental Medicine of the Siberian Branch of the Russian Academy of Sciences, Novosibirsk, Russia; Novosibirsk State University, Novosibirsk, Russia; Institute of Cytology and Genetics of the Siberian Branch of the Russian Academy of Sciences, Novosibirsk, Russia; Institute of Cytology and Genetics of the Siberian Branch of the Russian Academy of Sciences, Novosibirsk, Russia Novosibirsk State University, Novosibirsk, Russia; Institute of Cytology and Genetics of the Siberian Branch of the Russian Academy of Sciences, Novosibirsk, Russia Novosibirsk State University, Novosibirsk, Russia Kurchatov Genomic Center of ICG SB RAS, Novosibirsk, Russia

**Keywords:** metabolomics, amino acids, acylcarnitines, gene networks, genetic markers, Parkinson’s disease, vascular parkinsonism, neurodegeneration, dry plasma stains, biomarker, метаболомика, аминокислоты, ацилкарнитины, генные сети, генетический маркер, болезнь Паркинсона, сосудистый паркинсонизм, нейродегенерация, сухие пятна плазмы крови, биомаркер

## Abstract

Parkinson’s disease (PD) and vascular parkinsonism (VP) are characterized by similar neurological syndromes but differ in pathogenesis, morphology, and therapeutic approaches. The molecular genetic mechanisms of these pathologies are multifactorial and involve multiple biological processes. To comprehensively analyze the pathophysiology of PD and VP, the methods of systems biology and gene network reconstruction are essential. In the current study, we performed metabolomic screening of amino acids and acylcarnitines in blood plasma of three groups of subjects: PD patients, VP patients and the control group. Comparative statistical analysis of the metabolic profiles identified significantly altered metabolites in the PD and the VP group. To identify potential mechanisms of amino acid and acylcarnitine metabolism disorders in PD and VP, regulatory gene networks were reconstructed using ANDSystem, a cognitive system. Regulatory pathways to the enzymes converting significant metabolites were found from PD-specific genetic markers, VP-specific genetic markers, and the group of genetic markers common to the two diseases. Comparative analysis of molecular genetic pathways in gene networks allowed us to identify both specific and non-specific molecular mechanisms associated with changes in the metabolomic profile in PD and VP. Regulatory pathways with potentially impaired function in these pathologies were discovered. The regulatory pathways to the enzymes ALDH2, BCAT1, AL1B1, and UD11 were found to be specific for PD, while the pathways regulating OCTC, FURIN, and S22A6 were specific for VP. The pathways regulating BCAT2, ODPB and P4HA1 were associated with genetic markers common to both diseases. The results obtained deepen the understanding of pathological processes in PD and VP and can be used for application of diagnostic systems based on the evaluation of the amino acids and acylcarnitines profile in blood plasma of patients with PD and VP.

## Abstract (ru)

Болезнь Паркинсона (БП) и сосудистый паркинсонизм (СП) характеризуются схожими неврологическими синдромами, но различаются патогенезом, морфологией и терапевтическими подходами. Их молекулярногенетические механизмы многофакторны и задействуют множество биологических процессов. Для комплексного анализа патофизиологии этих заболеваний необходимо применение методов системной биологии и реконструкции генных сетей. В данном исследовании проведен метаболомный скрининг аминокислот и ацилкарнитинов в плазме крови трех групп испытуемых: пациентов с БП, пациентов с СП и контрольной группы. Сравнительный статистический анализ метаболомных профилей групп пациентов по сравнению с контролем определил значимо измененные уровни метаболитов при болезни Паркинсона и при сосудистом паркинсонизме. Для выявления потенциальных механизмов нарушения метаболизма аминокислот и ацилкарнитинов при БП и СП были реконструированы регуляторные генные сети с помощью когнитивной системы ANDSystem. Пути регуляции ферментов метаболизма значимых метаболитов были найдены для трех групп генетических маркеров: специфических для БП, специфических для СП, а также группы общих маркеров двух заболеваний. Сравнительный анализ молекулярно-генетических путей в генных сетях позволил выявить как специфические, так и общие для БП и СП молекулярные механизмы, ассоциированные с изменением метаболомного профиля. Обнаружены регуляторные пути, функция которых потенциально нарушена при этих патологиях. Специфическими для генетических маркеров БП оказались пути регуляции ферментов ALDH2, BCAT1, AL1B1 и UD11, а для генетических маркеров СП – пути регуляции ферментов OCTC, FURIN и S22A6. Регуляторные пути к ферментам BCAT2, ODPB и P4HA1 были связаны с общими для обоих заболеваний генетическими маркерами. Полученные результаты углубляют понимание патологических процессов при БП и СП и могут быть использованы для применения диагностических систем на основе оценки метаболомного профиля аминокислот и ацилкарнитинов в плазме крови пациентов с болезнью Паркинсона и сосудистым паркинсонизмом.

## Introduction

Parkinson’s disease (PD) and vascular parkinsonism (VP) are complex disorders characterized by bradykinesia, muscle rigidity, gait disturbances, and balance impairment in patients. PD is classified as a neurodegenerative disease, while VP, also known as “small vessel disease”, arises in the context of cerebrovascular diseases

In the pathogenesis of PD, disruptions in the nigrostriatal dopaminergic pathway play a crucial role, including depletion of dopamine reserves and neuronal loss in the pars compacta of substantia nigra (Alexander, 2004). Neurodegenerative processes in PD exhibit a distinct morphological staging of progression, beginning with the involvement of the olfactory bulb and the dorsal motor nucleus of the vagus nerve, eventually culminating in the critical loss of neurons in the substantia nigra pars compacta (Braak et al., 2003). This stepwise progression aligns with the gradual clinical manifestation of PD symptoms, starting from autonomic disturbances and advancing to core motor symptoms (bradykinesia, tremor, muscle rigidity) and cognitive deficits. The molecular mechanisms underlying PD are actively investigated within the scientific community. Known key factors include proteolytic stress, impaired energy metabolism in substantia nigra neurons, mitochondrial dysfunction (Levin et al., 2022), and the accumulation of alpha-synuclein (Rocha et al., 2018).

The mechanisms underlying vascular parkinsonism (VP) associated with cerebrovascular diseases (CVD) remain poorly understood. VP often arises in the context of CVD and chronic cerebral circulation disorders, leading to dysfunction of the neurogliovascular unit (Che Mohd Nassir et al., 2021). The symptoms of vascular parkinsonism develop more rapidly than in Parkinson’s disease and include lower-body-predominant bilateral parkinsonism, absence of tremor, pyramidal signs, and cognitive impairments (Vale et al., 2012). Unlike PD, dominated by proteolitic stress, mitochondrial dysfunction and the impairment energy metabolism of substantia nigra neurons (Levin et al., 2022), the pathogenesis of which involves the death of dopaminergic neurons and accumulation of alpha-synuclein (Rocha et al., 2018), VP is primarily driven by disturbances in microcirculation and hemodynamics. A key factor in VP development is small cerebral vessels lesion, often associated with a long history of arterial hypertension (Che Mohd Nassir et al., 2021) and diabetes mellitus (Thanvi et al., 2005). Chronic ischemia resulting from cerebrovascular disorders is accompanied by oxidative stress, inflammation, and mitochondrial dysfunction. These pathological processes lead to significant structural and functional changes in the neurogliovascular unit, including endothelial dysfunction, impaired blood-brain barrier permeability, and alterations in astrocytes and pericytes (Narasimhan et al., 2022). Ultimately, this results in white matter damage (leukoaraiosis) and the formation of multiple lacunar infarcts in strategically important areas of the basal ganglia (Zijlmans et al., 1995; Chen Y.-F. et al., 2014; Korczyn, 2015).

Among the common characteristics of these pathological processes are disruptions in the metabolism of lipids, amino acids, and energy molecules, highlighting their importance in the molecular mechanisms of these conditions. Amino acids and acylcarnitines are involved in numerous processes, including neurotransmitter biosynthesis and energy metabolism (Jones et al., 2010; Dalangin et al., 2020). Studies analyzing metabolomic profiles in Parkinson’s disease (PD) are available in the literature (Wuolikainen et al., 2016; Zhao et al., 2018; Ostrakhovitch et al., 2022), but the role of amino acids and acylcarnitines requires further investigation. It is also worth noting that, to date, we have not identified any metabolomic studies focused on vascular parkinsonism.

To study complex diseases such as Parkinson’s disease and parkinsonism, gene networks have been utilized. These networks allow for integration of knowledge and identification of regulatory mechanisms underlying pathologies at the molecular and genetic levels (Mercatelli et al., 2020). To date, studies on gene networks in Parkinson’s disease are presented, including protein-protein interaction networks of PD markers (George et al., 2019a; Tomkins, Manzoni, 2021), gene co-expression networks (George et al., 2019b), regulatory pathways (https://www.kegg.jp/entry/hsa05012), and others. In contrast to PD, studies on the molecular and genetic mechanisms of vascular parkinsonism based on gene networks are sparsely represented in the scientific literature (Chen Y. et al., 2022).

At the ICG SB RAS, the cognitive system ANDSystem was developed for reconstructing and analyzing gene networks using artificial intelligence methods (Demenkov et al., 2012; Ivanisenko V.A. et al., 2015, 2019; Ivanisenko T.V. et al., 2020, 2022). ANDSystem has been used for interpreting metabolomic (Rogachev et al., 2021; Ivanisenko V.A. et al., 2022, 2023) and proteomic (Pastushkova et al., 2013, 2019; Binder et al., 2014; Larina et al., 2015) data. Bioinformatics studies conducted with ANDSystem have expanded the understanding of molecular and genetic processes associated with the development of various diseases and the formation of comorbid conditions (Bragina et al., 2014, 2016, 2023; Saik et al., 2016, 2018, 2019; Zolotareva et al., 2019).

The aim of this study was a comparative analysis of the molecular and genetic mechanisms of PD and VP using the gene network reconstruction methods based on metabolomic screening of amino acids and acylcarnitines.

## Materials and methods

**Characteristics of patients groups. **The study included two groups of patients with confirmed diagnoses of Parkinson’s disease (PD) and vascular parkinsonism (VP), along with a control group. Differential diagnosis was based on MRI and clinical criteria. Blood samples were collected after discontinuation of L-DOPA (L-dihydroxyphenylalanine) treatment.

The PD group consisted of 9 patients (5 women, 4 men) with a mean age of 72.2 years (age range: 64–88 years). Inclusion criteria were: clinically confirmed PD, stage IV by Hoehn and Yahr, disease duration >5 years, and onset age 55–75 years; symptoms including bradykinesia, resting tremor or muscle rigidity, and response to L-DOPA therapy. The VP group included 9 patients (7 women, 2 men) with a mean age of 74.6 years (age range: 60–89 years). Inclusion criteria were: disease duration >3 years, MRI findings of multi-lacunar status and leukoaraiosis; symptoms such as lower-body-predominant parkinsonism, bilateral onset, and postural instability. The control group consisted of 17 conditionally healthy individuals (11 women, 6 men) with a mean age of 68 years (age range: 51–82 years). Background conditions in this group included chronic arterial hypertension without transient ischemic attacks or stroke and the absence of neurological symptoms.

**Collection of biological material and HPLC-MS/MS analysis. **Blood samples were collected from a peripheral vein during daytime, three hours after food intake, using 6 mL plasma tubes containing lithium heparin (68 IU) (Vacutainer, BD). Plasma preparation and the analysis of amino acids and acylcarnitines (the list of the analyzed metabolites is provided in Supplementary Material 1)1 were performed using the HPLC-MS/MS method as previously described (Kasakin et al., 2019). The analysis utilized an API 6500 QTRAP mass spectrometer (AB SCIEX, USA) coupled with an HPLC LC-20AD Prominence chromatograph (Shimadzu, Japan) equipped with an SIL-20AC autosampler (Shimadzu, Japan).


Supplementary Materials are available in the online version of the paper: https://vavilov.elpub.ru/jour/manager/files/Suppl_Makarova_Engl_28_8.xlsx


**Statistical analysis of experimental data.**For the statistical analysis of differences in metabolite levels across the metabolomic profiles of the studied groups, the Mann–Whitney and Kolmogorov–Smirnov tests were applied with a subsequent Benjamini–Hochberg procedure (False Discovery Rate, FDR) for multiple comparisons. Statistical calculations were performed using Python 3.11 with functions from the scipy.stats module. 

**Formation of lists of enzymes and genetic markers of PD and VP. **For each of the metabolites, the concentrations of which were statistically significantly altered in patient groups compared to the control group, lists of biosynthesis and degradation enzymes were made. The enzymes converting significant metabolites were extracted from the KEGG (Kanehisa, 2000) and HMDB (Wishart et al., 2022) databases

The lists of genetic markers for Parkinson’s disease and vascular parkinsonism were extracted from the MalaCards database (https://www.malacards.org/, accessed on: 25.01.2024) (Rappaport et al., 2014). The genetic markers of VP included protein-coding genes annotated in disease terms “Vascular Parkinsonism” and “Vascular Dementia”. The genetic markers for PD included protein-coding genes associated with the term “Parkinson’s Disease”.

**Gene networks reconstruction.** Gene networks reconstruction was performed using ANDVisio, a graphical user interface of the cognitive system ANDSystem (Ivanisenko V.A. et al., 2015). Regulatory pathways of four types were constructed according to the templates described in the Table. These templates allow to identify molecular genetic pathways including protein-protein interactions, regulation of protein activity, degradation, transport, proteolysis, and also gene expression regulation. The reconstruction of molecular genetic pathways regulating the enzymes that convert metabolites was carried out using the same templates for three sets of genetic markers (PD, VP, and common markers for both diseases).

**Table 1.**
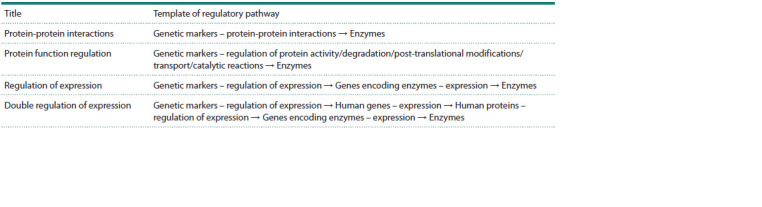
Templates of molecular genetic pathways regulating the biosynthesis and degradation enzymes of significant metabolites by genetic markers of PD, VP, or common to both diseases Notе. Genetic markers – proteins encoded by genetic markers (of PD, VP or common markers of both diseases); Enzymes – enzymes of conversion of significant metabolites; Enzyme genes – genes encoding enzymes of conversion of metabolomic markers.

## Results

Statistical analysis of metabolomic data

The statistical analysis of metabolomic data (Supplementary Material 1), aimed at identifying differences in metabolite levels between the PD and VP groups compared to the control group, revealed that out of 44 metabolites with measured concentrations, statistically significant differences (FDR < 0.05) were observed for 18 metabolites in PD and 21 metabolites in VP (Supplementary Material 2).

Both the PD and the VP group differed from the control group in the levels of four out of 14 analyzed amino acids: alanine, proline, isoleucine, and valine. Notably, methionine levels were significantly altered in the PD group but did not distinguish the VP group from the control. Among acylcarnitines, significant differences were identified for 13 metabolites shared between PD and VP (Supplementary Material 2). Specific acylcarnitines, the levels of which significantly differed only in the VP group compared to the control, included acylcarnitines C6, C10, C10:1, and Carnitine.

Reconstruction and analysis of gene networks

To investigate the molecular genetic mechanisms potentially contributing to the altered metabolomic profiles in PD and VP, we utilized the gene network approach. Gene networks enabled the integration of knowledge about the molecular interactions of metabolites with known genetic markers of PD and VP. Genetic markers were defined as genes associated with PD and VP according to the MalaCards database (Rappaport et al., 2014). Lists of 84 genetic markers for Parkinson’s disease and 60 markers for vascular parkinsonism are provided in Supplementary Material 3. The intersection of the genetic marker lists for PD and VP showed that 22 genetic markers were shared between these diseases.

To study the role of genetic markers in the regulation of enzymes involved in the conversion of significant metabolites, we applied the gene network approach using the ANDVisio software (Ivanisenko V.A. et al., 2019). This method is based on the automated reconstruction of regulatory molecular genetic pathways using templates specified in the queries to ANDVisio (see the Table).

We analyzed the regulatory pathway templates that start with proteins encoded by genetic markers specific to PD, to VP, and also markers common to both diseases. The lists of these proteins were used as input data for the “Pathway Wizard” module of the ANDVisio software. The regulatory pathways end with the enzymes of biosynthesis and degradation of metabolites identified as significant in the statistical analysis. The lists of enzymes used for the analysis are provided in Supplementary Material 4. The pathways also include intermediate participants (human proteins) that link genetic markers to enzymes. These intermediate proteins were not explicitly specified in the input data, as the software automatically identified such mediators. The regulatory pathways accounted for major types of molecular genetic interactions, including gene expression regulation, protein-protein interactions, and regulation of protein activity, degradation, transport, and catalytic reactions. Illustrations of the gene networks are provided in Supplementary Materials 5 and 6. The number of regulatory connections to each enzyme originating from PD, VP, and shared genetic markers is shown in Supplementary Materials 7–9.

**Fig. 1.**
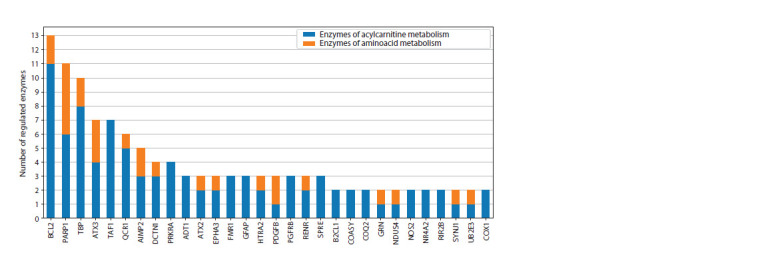
Distribution of the number of regulatory pathways in the gene network from PD genetic markers to the enzymes of amino acid and acylcarnitine metabolism.

**Fig. 2.**
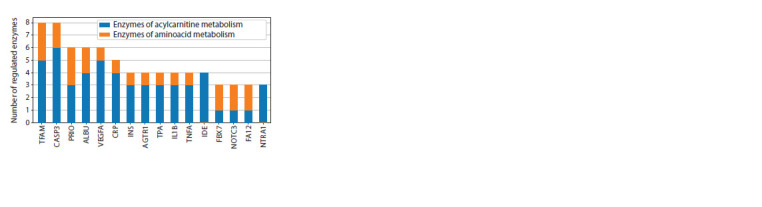
Distribution of the number of regulatory pathways in the gene network from VP genetic markers to the enzymes of amino acid and acylcarnitine metabolism.

Histograms illustrating the distribution of regulatory connections among participants of the reconstructed gene networks are shown in Figures 1 and 2. In the histograms, the X axis represents the names of genetic markers, while the Y axis shows the number of regulatory pathways realized through molecular genetic interactions from the genetic markers (PD, VP, and shared markers) to the enzymes involved in reactions with significant metabolites

The genetic markers of Parkinson’s disease BCL2, TBP, and TAF1 exert greater regulatory influence on acylcarnitine metabolism enzymes, while PARP1 equally affects enzymes involved in both amino acid and acylcarnitine metabolism (Fig. 1). The genetic markers of vascular parkinsonism such as TFAM, CASP3, ALBU, and VEGFA have a stronger regulatory influence on acylcarnitine metabolism enzymes, whereas FBX7, NOTC3, and FA12 predominantly affect amino acid metabolism enzymes (Fig. 2).

**Fig. 3.**
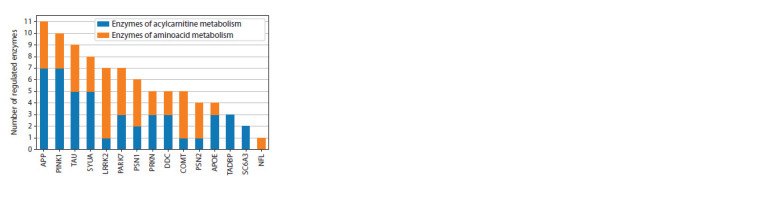
Distribution of the number of regulatory pathways in the gene network from genetic markers shared between PD and VP to the enzymes of amino acid and acylcarnitine metabolism.

Notably, the genetic markers shared between PD and VP participate equally in regulating enzymes involved in amino acid and acylcarnitine metabolism (Fig. 3). The genetic marker LRRK2, according to the gene networks, exerts a greater influence on the regulation of amino acid metabolism.

**Fig. 4.**
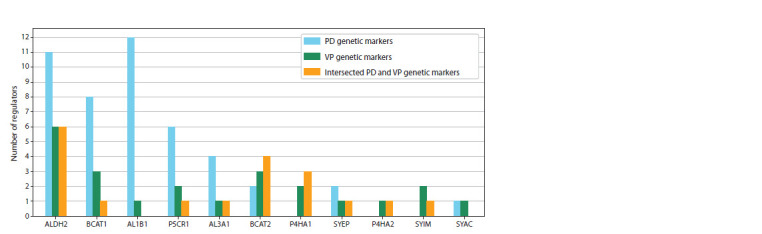
Distribution of the number of regulatory pathways in the gene networks from genetic markers (PD, VP and shared between PD and VP) to the enzymes of amino acid metabolism.

**Fig. 5.**
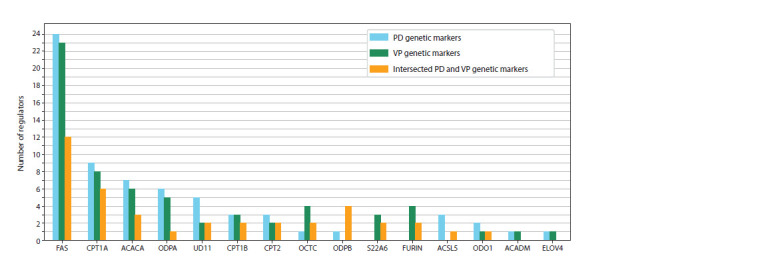
Distribution of the number of regulatory pathways in the gene networks from genetic markers (PD, VP and shared between PD and VP) to the enzymes of acylcarnitine metabolism

For some enzymes involved in metabolite conversion, the levels of which significantly differed in PD and VP patients compared to the control group, the regulatory pathways originating from genetic markers of PD, VP, and common genetic markers demonstrated varying quantitative proportions. Histograms depicting the number of regulatory impacts from groups of genetic markers to enzymes of amino acid and acylcarnitine metabolism were based on the gene networks (Supplementary Materials 5 and 6) and are shown in Figures 4 and 5.

The regulatory pathways from PD genetic markers are more prominent for the enzymes ALDH2, BCAT1, AL1B1, and P5CR1, while pathways to BCAT2 and P4HA1 originate more from the genetic markers shared between PD and VP (Fig. 4). Among the enzymes of acylcarnitine metabolism, fatty acid synthase (FAS) is subject to the most significant regulatory influence (Fig. 5). For enzymes FAS, ODPA (PDHA1), and ACACA (ACC1), regulatory pathways are implemented by genetic markers specific to both PD and VP. However, in regulation of the enzyme ODPB, shared genetic markers play a more prominent role.

Thus, the metabolomic analysis identified 5 amino acids and 17 acylcarnitines with significantly altered concentrations in the PD and VP patient groups compared to the control group (Supplementary Material 2). The gene network approach enabled the reconstruction and analysis of regulatory molecular genetic pathways from PD and VP genetic markers to the enzymes involved in amino acid and acylcarnitine metabolism

## Discussion

Specific and non-specific markers of PD and VP

Our results showed that the lists of 18 and 21 significant metabolites for PD and VP, respectively, had an overlap in 17 common metabolites, which could be considered as nonspecific markers for differential diagnosis. The metabolomic analysis thus provided a limited insight into distinguishing between Parkinson’s disease and vascular parkinsonism. We hypothesized that while potential metabolomic markers overlap for PD and VP, the molecular mechanisms underlying their metabolic disruptions may differ between the two diseases. It is known that genetic markers play a substantial role in pathological processes. In this regard, the genetic markers may also influence the metabolism of the potential PD and VP markers (amino acids and acylcarnitines) identified in our study.

To test this hypothesis, we reconstructed the gene networks describing regulatory connections from the genetic markers of these diseases to the enzymes involved in the biosynthesis and degradation of significant metabolites. The analysis revealed that disease-specific genetic markers actively regulate enzyme functions and the expression of their encoding genes (Supplementary Materials 5 and 6). Genetic markers were grouped into three categories: specific to PD, specific to VP, and shared between the two diseases. To identify the specific molecular mechanisms of disrupted metabolism regulation in PD and VP, we analyzed the regulatory pathways starting from disease-specific genetic markers. Meanwhile, the pathways involving genetic markers shared between PD and VP were hypothesized to define the mechanisms underlying common metabolomic profile disruptions. In the reconstructed gene networks (Supplementary Materials 5 and 6), we highlighted the regulatory pathways involving enzymes previously studied in the context of Parkinson’s disease and vascular parkinsonism

The gene networks of regulation of the enzymes of amino acids metabolism

**Fig. 6.**
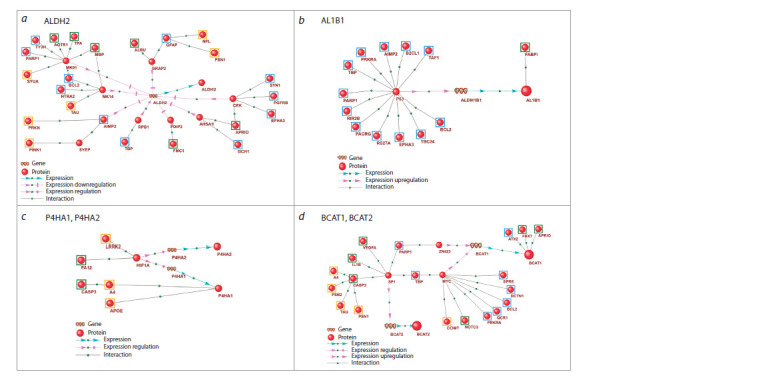
Gene networks of regulation of the enzymes involved in amino acid metabolism by genetic markers of PD, VP and common markers of PD and VP. The genetic markers are framed: PD (blue frames), VP (green frames), common markers of PD and VP (orange frames).

ALDH2 (aldehyde dehydrogenase 2) was identified among the enzymes with the highest number of regulatory connections from PD and VP genetic markers (Fig. 6a). ALDH2 participates in the metabolism of proline, alanine, and fatty acids and is a key enzyme involved in metabolizing aldehydes and cytotoxic metabolites. In the brain, ALDH2 plays a crucial role in preventing “aldehyde load” – the accumulation of aldehydes that, under oxidative stress, can bind to lipids, nucleic acids, and proteins, causing neurotoxic effects (Chen C.-H. et al., 2016). Studies on the association of aldehyde dehydrogenases with PD have shown increased mitochondrial ALDH2 activity in the striatum of PD patients (Michel et al., 2014). ALDH2 may protect neurons from the toxic effects of dopamine metabolites (Chiu et al., 2015), and enhanced ALDH2 activity has been shown to restore neuronal function impaired by hypoxia (Lin et al., 2022).

The enzyme AL1B1 (mitochondrial aldehyde dehydrogenase X) had the highest number of regulatory connections from PD genetic markers in the gene network (Fig. 6b). AL1B1 is involved in proline, alanine, and fatty acid metabolism and plays a substantial role in acetaldehyde detoxification and neurotransmitter metabolism (Shortall et al., 2021). It was suggested that AL1B1 deficiency identified in the brain is associated with Parkinson’s disease progression (Grünblatt, Riederer, 2016; Odongo et al., 2023). AL1B1 deficiency may lead to the accumulation of aldehydes such as 4-hydroxy-2- nonenal (4-HNE), which can impair mitochondrial function, induce alpha-synuclein aggregation, and trigger neuroinflammation and apoptosis (Wey et al., 2012; Grünblatt, Riederer, 2016).

According to the gene network analysis, the enzymes P4HA1 and P4HA2 were more strongly regulated by the genetic markers shared between PD and VP (Fig. 6c). P4HA (prolyl 4-hydroxylase alpha) enzymes catalyze the formation of 4-hydroxyproline, essential for the correct folding of procollagen chains (Song et al., 2023). Additionally, P4HA1 is known to participate in post-ischemic angiogenesis (Xu et al., 2024).

The enzymes BCAT1 and BCAT2 catalyze the reversible transamination of branched-chain amino acids (BCAA) with alpha-ketoglutarate to form corresponding branched-chain alpha-keto acids and glutamate. Our metabolomic analysis revealed elevated levels of BCAAs, such as valine and isoleucine, in PD and VP patients. Based on our reconstructed gene networks, BCAT1 was one of the enzymes highly regulated by PD genetic markers, while regulatory connections to BCAT2 predominantly originated from shared PD and VP markers (Fig. 6d ). Defective BCAA metabolism, including BCAT1 disruptions, is associated with key PD features, including motor dysfunction and neurodegeneration (Yao et al., 2018; Sohrabi et al., 2021). In Parkinson’s disease C. elegans models, knockdown of bcat1 led to depletion of tricarboxylic acid cycle metabolites and mitochondrial hyperactivity, resulting in oxidative damage to neurons (Mor et al., 2020). Furthermore, a genome-wide association meta-analysis has linked PD to genes encoding BCAA metabolism enzymes (Nalls et al., 2014). Disruptions in BCAA metabolism enzymes have also been observed in vascular dementia. Increased mRNA expression of cytosolic and mitochondrial BCAT was found in cortical samples from patients with vascular dementia, possibly protecting cells from the neurotoxic effects of excess glutamate (Ashby et al., 2017).

The gene networks of regulation of the enzymes of acylcarnitines metabolism

**Fig. 7.**
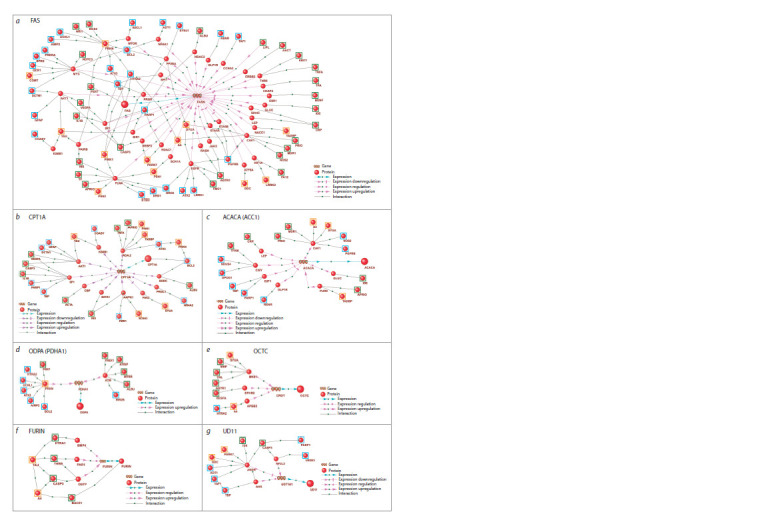
Gene networks of regulation of the enzymes involved in acylcarnitines metabolism by genetic markers of PD, VP and common markers of PD and VP. The genetic markers are framed: PD (blue frames), VP (green frames), common markers of PD and VP (orange frames).

In both patient groups, we identified alterations in the acylcarnitine profile, which plays a critical role in the cellular energy metabolism. As acylcarnitines are the primary carriers of fatty acids to the inner mitochondrial membrane, their metabolism is closely linked to fatty acid metabolism. Fatty acid synthase (FAS) catalyzes the elongation of fatty acids starting from acetyl-CoA and malonyl-CoA. In the gene network regulating acylcarnitine metabolism enzymes, the FASN gene had the highest number of “expression regulation” connections from genetic markers of PD and VP (Fig. 7a). Notably, the genetic marker PINK1, associated with mitochondrial dysfunction in PD (Narendra et al., 2010), has been implicated in this pathway. Mutations in PINK1 lead to its deficiency in PD (Valente et al., 2004). It has been shown that FAS repression in PINK1- mutant models restores mitochondrial metabolic processes and reduces palmitate levels (Vos et al., 2017). Additionally, FAS is known to play a role in central nervous system myelination and remyelination processes (Dimas et al., 2019).

The gene network analysis revealed numerous regulatory connections to CPT1 (carnitine palmitoyltransferase 1) from the genetic markers specific to both PD and VP (Fig. 7b). CPT1 is a transporter protein located on the outer mitochondrial membrane; CPT1 exists in three isoforms in mammalian cells: CPT1A, CPT1B, and CPT1C. CPT1A is more specific to lipogenic tissues (e. g., liver), while CPT1B predominates in tissues with high fatty acid oxidation capacity (e. g., heart and skeletal muscle), and CPT1C is predominantly expressed in neuronal tissue (Wang Muyun et al., 2021). CPT1 enzymes catalyze the transfer of acyl-CoA groups (chain lengths C12– C18) to L-carnitine, forming acylcarnitines (Schlaepfer, Joshi, 2020). Inhibition of lipid metabolism regulated by CPT1A in mouse models of Parkinson’s disease has shown promising results, improving motor and sensorimotor functions (Trabjerg et al., 2023). CPT1 has also been implicated in the development of insulin resistance, a condition associated with impaired function of substantia nigra in the brain (Virmani et al., 2015). In early-stage PD patients, reduced levels of longchain acylcarnitines (C14–C18) were identified, potentially associated with CPT1 deficiency (Saiki et al., 2017).

Regulatory connections to the enzymes ACC1 and ODPA (PDHA1) were characteristic for both PD and VP (Fig. 7c, d ). ACC1 (acetyl-CoA carboxylase 1, ACACA) is the rate-limiting enzyme in de novo fatty acid synthesis, converting acetyl-CoA to malonyl-CoA (Wang Y. et al., 2022). In Parkinson’s disease models, interaction of phosphorylated alpha-synuclein and ACC1 has been associated with low ATP levels, oxidative stress, and mitochondrial dysfunction (Grassi et al., 2018). PDHA1 (pyruvate dehydrogenase E1 alpha, ODPA) is a key component of the complex that catalyzes the decarboxylation of pyruvate to acetyl-CoA (Børglum et al., 1996). Under stress conditions, PDHA1 suppression enables astrocytes to rely on anaerobic glycolysis, increasing lactate consumption by neurons, conserving glucose, and protecting against oxidative stress (de Holanda Paranhos et al., 2024). Thus, PDHA1 acts as a mediator between cytosolic glycolysis and mitochondrial oxidative phosphorylation (Pavlú-Pereira et al., 2023). Research by Miki Y. et al. (2017) demonstrated that PDHA1 is a component of Lewy bodies in idiopathic PD and PARK14-linked parkinsonism (a familial PD form). Additionally, reduced PDHA1 protein levels have been observed in brain regions such as the striatum and substantia nigra in idiopathic PD patients.

According to the analysis of gene networks, regulatory connections to the enzymes OCTC and FURIN were found to be more specific to VP genetic markers (Fig. 7e, f ). OCTC (peroxisomal carnitine octanoyltransferase), encoded by the CROT gene, is involved in the transport of medium- and long-chain acyl-CoA from peroxisomes, which are critical for β-oxidation of fatty acids. OCTC has been associated with calcification of arterial smooth muscle cells, as high OCTC levels were detected near calcified plaque areas (Okui et al., 2021).

Furin (PACE) is a serine convertase involved in atherogenesis. Increased furin activity is associated with cardiovascular disease progression (Wichaiyo et al., 2024), and its inhibition has been shown to slow atherosclerotic lesion progression in mice (Yakala et al., 2019). Furin also affects neuronal tissue by promoting the conversion of brain-derived neurotrophic factor (BDNF) from pro-BDNF to its mature form, potentially influencing neurodegenerative diseases (Wang Mingyue et al., 2021). Furin inhibitors may prevent neuronal damage induced by NMDA signaling (Yamada et al., 2018) and facilitate the conversion of pro-nerve growth factor (pro-NGF) to β-NGF, which influences vascular smooth muscle cells (Urban et al., 2013). Studies on the Parkinson’s disease models showed that furin modulates disease progression. For instance, knockdown of Furin1 in D. melanogaster reduced dopaminergic neuron loss caused by mutations in Lrrk2 (Maksoud et al., 2019). Furthermore, furin is required for cap-dependent LRRK2 translation, impacting postsynaptic signaling (Penney et al., 2016).

Regulatory connections to the enzyme UD11 were predominantly driven by PD genetic markers (Fig. 7g). UDP-glu curonosyltransferases are enzymes involved in detoxification by glucuronidating substrates, facilitating their excretion (Tukey, Strassburg, 2000). While these enzymes are understudied in the context of PD and VP, the link of UDPglucuronosyltransferase 1A9 genotype to adverse reactions to catechol-O-methyltransferase inhibitors in PD patients was reported (Ferrari et al., 2012).

The altered metabolomic profiles of amino acids and acylcarnitines in PD and VP may result from distinct molecular genetic mechanisms. In this study, the regulatory pathways specific to PD included the enzymes ALDH2, BCAT1, AL1B1, and UD11. The pathways specific to VP were identified for OCTC, FURIN, and S22A6. For genetic markers shared by PD and VP, regulatory influences were prominent on the enzymes BCAT2, ODPB, and P4HA1. The gene networks analysis for both PD and VP revealed disruptions in lipid metabolism, valine and isoleucine pathways, and mechanisms associated with oxidative stress and mitochondrial dysfunction.

## Conclusion

To identify disease-specific molecular genetic mechanisms, we reconstructed gene networks describing the regulation of enzymes involved in the metabolism of potential PD and VP markers identified by HPLC-MS/MS, including several amino acids (alanine, proline, valine, isoleucine, methionine) and 17 acylcarnitines. A comparative analysis of regulatory pathways within these networks revealed both specific and non-specific molecular mechanisms associated with the altered metabolomic profiles of these pathologies. The results obtained highlight the molecular genetic distinctions between PD and VP and may be useful for the development and application of diagnostic systems based on plasma metabolomic profiles of amino acids and acylcarnitines. Notably, this study was the first to apply the gene network analysis to the metabolomic profiles of amino acids and acylcarnitines in patients with vascular parkinsonism and Parkinson’s disease, representing a significant step forward in the comparative investigation of these disorders.

## Conflict of interest

The authors declare no conflict of interest.
